# Efficacy and safety of microneedling with topical insulin compared with placebo in the treatment of atrophic scars: a prospective study and literature review

**DOI:** 10.1093/skinhd/vzaf032

**Published:** 2025-04-23

**Authors:** Bushra Karkour, Sedra Abu Ghedda, Rashed Aljundi, Sedra Sheikh Debs, Reem Farwati, Silva Ishkhanian

**Affiliations:** Department of Dermatology and Venereology, Aleppo University Hospital, Aleppo, Syria; Faculty of Medicine, University of Aleppo, Aleppo, Syria; Faculty of Medicine, University of Aleppo, Aleppo, Syria; Faculty of Medicine, University of Aleppo, Aleppo, Syria; Faculty of Medicine, University of Aleppo, Aleppo, Syria; Department of Dermatology and Venereology, Aleppo University Hospital, Aleppo, Syria

## Abstract

**Background:**

Atrophic scars, which affect many individuals and arise from various causes, currently lack a standardized treatment protocol. Recent studies suggest that combining microneedling with topical insulin (TI) may offer a promising new approach to improving the appearance of these scars.

**Objectives:**

To evaluate the efficacy and safety of microneedling combined with TI for treating atrophic scars caused by acne, cutaneous leishmaniasis, striae alba and postoperative wounds. Additionally, we aimed to assess the impact of treatment on improving quality of life.

**Methods:**

A total of 158 patients with various types of atrophic scars were divided into two groups: one received microneedling with TI and the other received microneedling with a placebo. Each participant underwent 12 monthly sessions, followed by a 1-year follow-up to assess long-term outcomes. Primary outcomes were measured via the Goodman and Baron qualitative grading system and acne scarring grading system, along with patient satisfaction and Dermatology Life Quality Index scores.

**Results:**

A statistically significant improvement was observed in the TI group, as reported by patients and clinicians, especially regarding postacne and postleishmaniasis scars (*P* = 0.001). The improvement in quality of life was most pronounced in the postacne group (*P* = 0.002).

**Conclusions:**

This study suggests that microneedling, when combined with TI, is a safe and effective standalone treatment for atrophic scars resulting from various causes. Additionally, this approach may enhance the quality of life for patients with this condition. However, further research with larger sample sizes is needed to validate these findings.

## Teaser

This prospective study evaluated the efficacy and safety of microneedling combined with topical insulin for treating various atrophic scars, including those from acne and cutaneous leishmaniasis. Results showed significant improvements in scar appearance and patient quality of life, particularly in post-acne scars (*P* = 0.001 and *P* = 0.002, respectively). These findings suggest that this combined treatment is a promising and effective option for atrophic scars, warranting further research with larger sample sizes to confirm the results.

**What is already known about this topic?**

- Microneedling combined with topical treatments may offer a promising option for addressing postacne atrophic scars; however, larger studies are necessary to validate its effectiveness.

**What does this study add?**

- This study revealed that microneedling with topical insulin can serve as an effective standalone treatment for atrophic scars resulting from acne, cutaneous leishmaniasis, striae alba and surgical wounds.

Atrophic scars are characterized primarily by widespread skin atrophy, resulting in a reduction in skin cells within the epidermis. Clinically, these scars manifest as a loss of normal dermal tissue.^1^ The most prevalent cause of atrophic scars worldwide is acne, which can produce three distinct types of scars: ice pick, boxcar and rolling scars. Other common causes include dermal stretching, known as striae alba, postoperative wounds and cutaneous leishmaniasis, which is common in the Middle East region.

Currently, there is no single, definitive treatment for atrophic scars. However, several combination therapies are available, which can pose challenges for patients because of the need for long-term adherence, associated costs and potential side-effects. Commonly used treatments include dermabrasion, subcision, punch techniques, chemical peels, tissue augmentation and lasers.^2^

Microneedling is also an effective method for treating scars, particularly when used alongside topical treatments.^3^ This technique involves controlled penetration of the skin via ultrafine needles, which stimulate collagen production and facilitate the transdermal delivery of medications.^3^ Insulin is a powerful contributor to the wound healing process and promotes fast recovery across different types of wounds.^4^ In addition to its role in managing re-epithelialization and inflammatory responses in wound tissues, insulin also has angiogenic properties that enhance healing.^4^ When applied topically, insulin stimulates collagen production and encourages the formation of new blood vessels in healing tissues by promoting the synthesis of transforming growth factor β1 and vascular endothelial growth factor.^5^

Recent studies have suggested that microneedling combined with topical insulin (TI) may be effective for treating atrophic scars, particularly those resulting from acne.^6^ However, these studies often face limitations, including small sample sizes and short follow-up periods, which constrains their findings.^6^ In this prospective study, we aimed to evaluate the efficacy and safety of microneedling with TI compared with those of a placebo in the treatment of various types of atrophic scars, including postacne scars, striae alba, cutaneous leishmaniasis and scars resulting from postoperative wounds. Our follow-up period extended to 1 year, allowing for a comprehensive assessment of treatment outcomes.

## Materials and methods

### Participants

Participants included patients who visited the dermatology outpatient clinics at Aleppo University Hospital in Aleppo, Syria, between July 2021 and September 2023.

Inclusion criteria were facial atrophic scars; postoperative atrophic scars; postleishmanial atrophic scars; and striae alba. The exclusion criteria were active infectious lesions or infectious lesions in the area to be treated; history of keloid formation; bleeding disorders; diabetes or glucose intolerance; pregnancy or lactation; dermatological disorders with isomorphic reactions; and refusal to participate in the study.

### Study design

**Figure 1:**
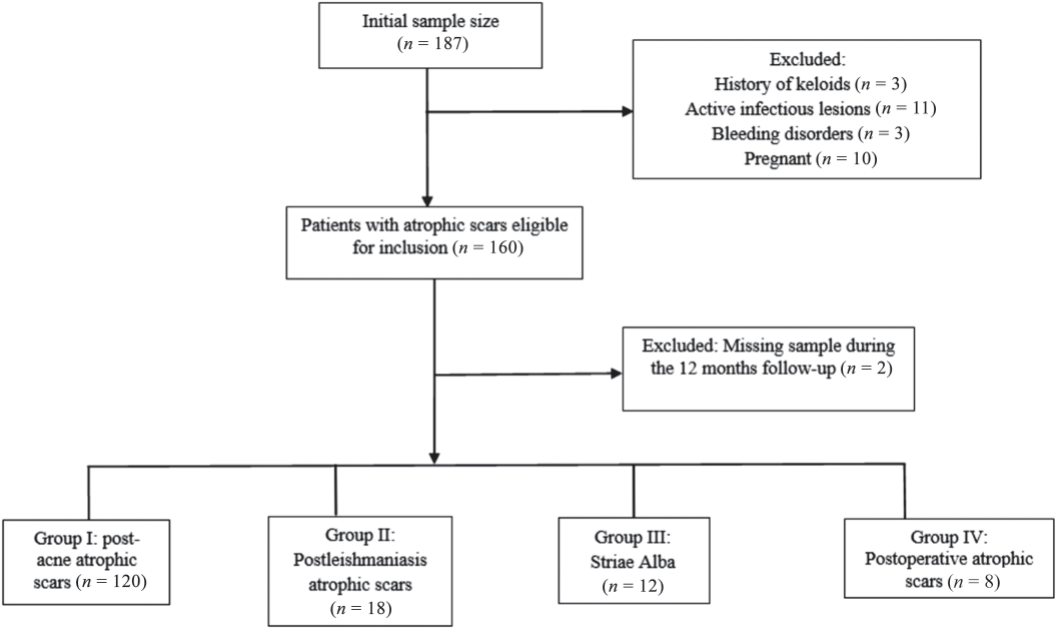
STROBE flowchart.

In this prospective cohort study, patients were categorized into four distinct groups (groups I–IV), as shown in Figure 1.^7^ Each group was further divided into two subgroups: patients in subgroup A received TI with microneedling, and those in subgroup B received a placebo with microneedling.

In Group I (postacne atrophic scars), subgroup A included 100 patients (63.3%) and subgroup B included 20 patients (12.7%); in group II (postleishmaniasis atrophic scars), subgroups A and B both included 9 patients (5.7%) each; in group III (striae alba), subgroups A and B both comprised 6 patients (3.8%); and in group IV (postoperative atrophic scars), subgroups A and B both included 4 patients (2.5%) each.

### Procedure

All patients were diagnosed clinically, and their atrophic scars were assessed via the Goodman and Baron grading system. Prior to the procedure, all patients underwent laboratory testing, including a complete blood count, prothrombin time, activated partial thromboplastin time, fasting glucose and haemoglobin A1c levels.

Initially, a topical anaesthetic was applied for 1 h, after which the area was sterilized. Microneedling was then performed via a 24 G disposable needle-tipped dermapen. The skin was stretched with one hand while the other hand guided the instrument in a direction perpendicular to the stretching force, employing a vibrating, stamp-like motion until pinpoint bleeding occurred. The needle depth was adjusted based on the treatment area: 0.75 mm for the forehead, 2.5 mm for the cheeks and striae alba and 1 mm for the chin.

During the treatment, the blood was wiped away with sterile saline solution to prevent encrustation. A solution of human actrapid insulin (100 IU mL^−1^) was applied to patients in subgroups A at a dose of 40 units, whereas patients in subgroups B received saline solution. After 1 h, the face was cleansed, and Panthenol (Revuele Beauty & Care, Sofia, Bulgaria) and Fucidin® cream (LEO Pharma, Ballerup, Denmark) were applied.

Patients were advised to avoid washing their faces for 24 h and to refrain from sun exposure or any heat sources for 2 days. Additionally, they were instructed to avoid the use of exfoliants and irritating washes for 1 week. An anti­biotic cream was prescribed for post-treatment care. The treatment sessions were conducted once a month for a total of 12 months (12 sessions).

For sun protection after each session, patients were recommended to apply a sunscreen with a protection factor of 30 or higher. Patients with a history of herpes simplex infection were prescribed prophylactic aciclovir. Throughout the 12-month period, patients followed a strict regimen to avoid any additional procedures and the use of topical tretinoins to prevent irritation and worsening of side-effects.

### Response assessment

Patient responses were evaluated at baseline and at the final visit via the Goodman and Baron qualitative and acne scarring grading systems.^8^ Improvement by one degree on the scale was assigned a score of 1, whereas a two-degree improvement received a score of 2. A response score of 2 or higher indicates a good response, a score between 1 and 2 indicates an average response, and a score below 1 signifies a poor response. All assessments were performed by three clinicians separately, and the median response was recorded to minimize bias.

### Patient satisfaction

Patients rated their perceived improvement on a scale of 1–10, with 1 being minimal improvement and 10 being maximal improvement. A score of 6 or higher was considered a good response, a score of 4–6 was considered an average response and a score of less than 4 was considered a poor response. Additionally, the Dermatology Life Quality Index was used at baseline and at the final visit.^9^ The response was evaluated on the basis of the impact of atrophic scars on daily life and was categorized as minimal (score of 2–5), moderate (score of 6–10) or severe (score of 11–30).

### Assessment of side-effects

Side-effects, including the following, were assessed at each visit: burning sensation, erythema, hyperpigmentation, induced acne, viral infections, bacterial infections, bruising, dryness and hypoglycaemia.

### Statistical analysis

Statistical analyses were performed with SPSS version 22 (IBM, Armonk, NY, USA). The significance level was established at a *P*-value of 0.05. Nominal variables were characterized via frequency distributions, whereas quantitative variables were summarized as mean (SD) .

To assess differences in the frequency of categorical variables, Fisher’s exact test was used. Independent samples *t*-tests were used to compare the mean values of continuous variables between two groups. For noncontinuous variables, nonparametric tests were applied to compare distributions within the same group or across different groups.

## Results

### Demographic data

Information regarding age, sex, Fitzpatrick skin type, previous treatments and duration of scars is shown in Table 1.

**Table 1:** Participant characteristics (*n* = 158)

Type of atrophic scar	Subgroup	Age (years), mean (SD)	Scar duration		Sex		Fitzpatrick skin type			Previous treatments
			<1 year	>1 year	Female	Male	II	III	IV	
Acne scars	A (*n* = 100)	26.13 (6.12)	68 (68)	32 (32)	91 (91)	9 (9)	20 (20)	60 (60)	20 (20)	65 (65)
	B (*n* = 20)	25.41 (5.22)	6 (30)	14 (70)	3 (15)	17 (85)	0	15 (75)	5 (25)	10 (50)
Leishmaniasis scars	A (*n* = 9)	17.66 (2.18)	4 (44)	5 (56)	7 (78)	2 (22)	0	6 (67)	3 (33)	2 (22)
	B (*n* = 9)	19 (3.14)	5 (56)	4 (44)	3 (33)	6 (67)	0	7 (78)	2 (22)	2 (22)
Striae alba	A (*n* = 6)	35.16 (4.18)	2 (33)	4 (67)	6 (100)	0	3 (50)	2 (33)	1 (17)	4 (67)
	B (*n* = 6)	34.66 (4.73)	2 (33)	4 (67)	6 (100)	0	2 (33)	2 (33)	2 (33)	3 (50)
Postoperative scars	A (*n* = 4)	22.25 (2.48)	3 (75)	1 (25)	3 (75)	1 (25)	1 (25)	2 (50)	1 (25)	1 (25)
	B (*n* = 4)	23.5 (2.63)	2 (50)	2 (50)	3 (75)	1 (25)	2 (50)	1 (25)	1 (25)	2 (50)

Data are presented as *n* (%) unless otherwise stated.

### Postacne atrophic scar group (group I)

With respect to the period without acne treatment, 70 patients (70%) in subgroup A had not received treatment for less <1 year, whereas the remaining 30 had been untreated for >1 year. In subgroup B, eight patients (40%) reported no treatment for <1 year.

In subgroup A, 60 patients (60%) reported a family history of postacne atrophic scars, whereas in subgroup B, 10 patients (50%) reported a similar family history. The cheeks were the most common scar site in both groups (85% in subgroup A and 75% in subgroup B) followed by the forehead and chin.

In subgroup A, the most prevalent type of scar was rolling scars, which were observed in 60 patients (60%). This was followed by boxcar scars in 33% and icepick scars in 7%. Similarly, in subgroup B, rolling scars were the most common, affecting 55% of patients, while boxcar scars affected 35% and icepick scars 10%.

#### Response assessment

In subgroup A, 50 of 100 patients (50%) had a good response, 40 (40%) had an average response and 10 (10%) had a poor response. In subgroup B, 5 of 20 patients (25%) had a good response, 10 (50%) had an average response and 5 (25%) had a poor response. Overall, 55 of 120 patients (45.8%) rated their response as good, 45 (37.5%) as average and 20 (16.7%) as poor.

**Figure 2:**
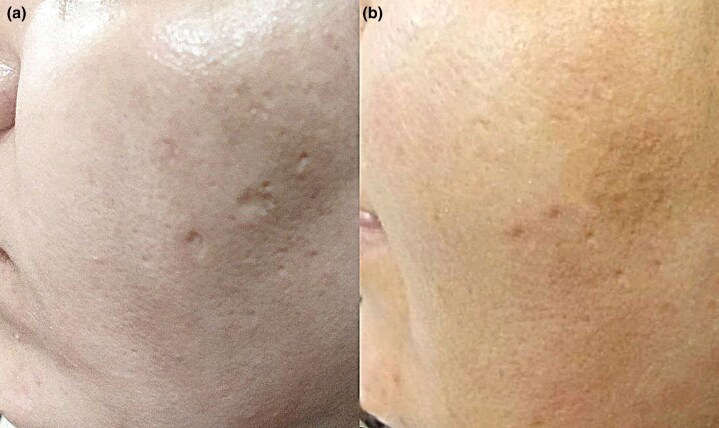
Postacne atrophic scars. (a) Before and (b) after 12 months of treatment with topical insulin a good response was observed.

**Figure 3:**
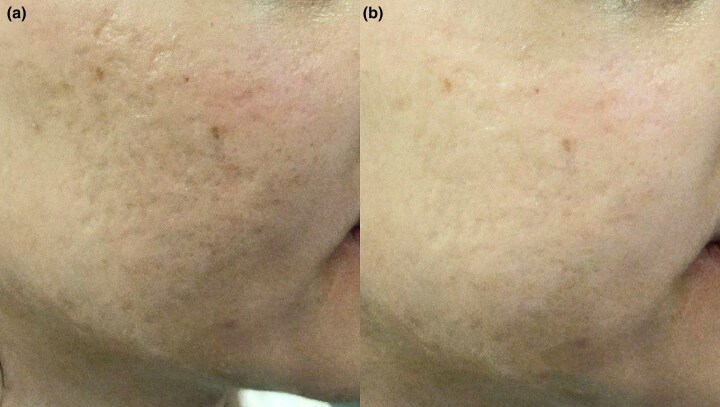
Postacne atrophic scars. (a) Before and (b) after 12 months of treatment with topical insulin, a moderate response was observed.

Fisher’s exact test revealed a significant difference between the two groups (*P* = 0.001) (Figures 2, 3).

### Postleishmaniasis scar group (group II)

In subgroup A, five of nine patients (56%) achieved a good response, three (33%) achieved an average response and one (11%) achieved a poor response. In subgroup B, two of nine patients (22%) had a good response, three (33%) had an average response and four (44%) had a poor response.

#### Response assessment

**Figure 4:**
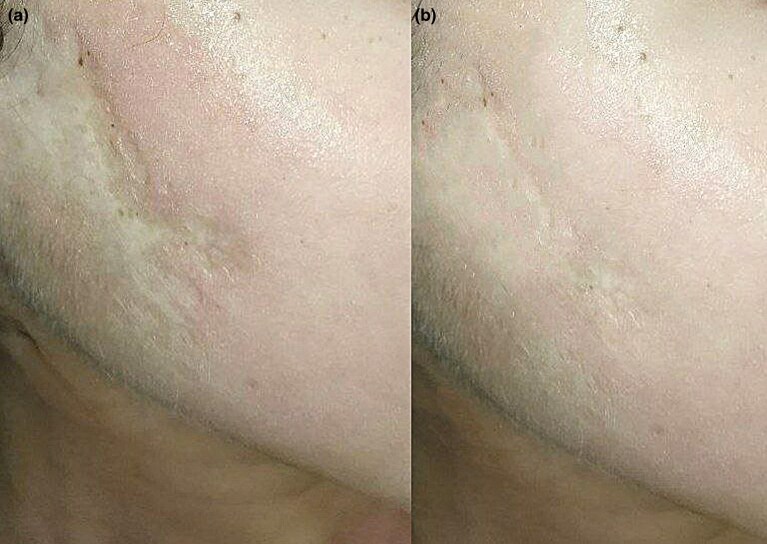
Postleishmaniasis atrophic scars. (a) Before and (b) after 12 months of treatment with topical insulin, a good response was observed.

**Figure 5:**
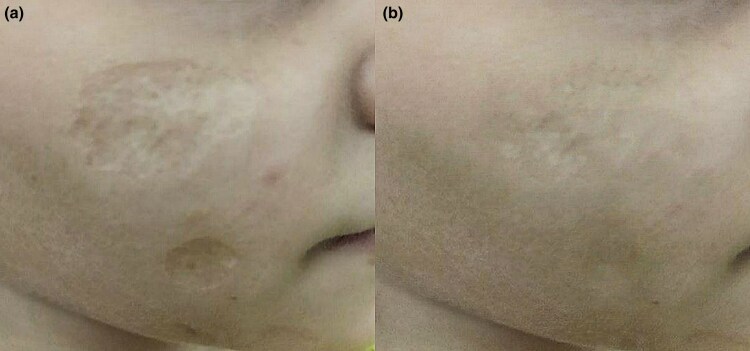
Postleishmaniasis atrophic scars. (a) Before and (b) after 12 months of treatment with topical insulin, a good response was observed.

**Figure 6:**
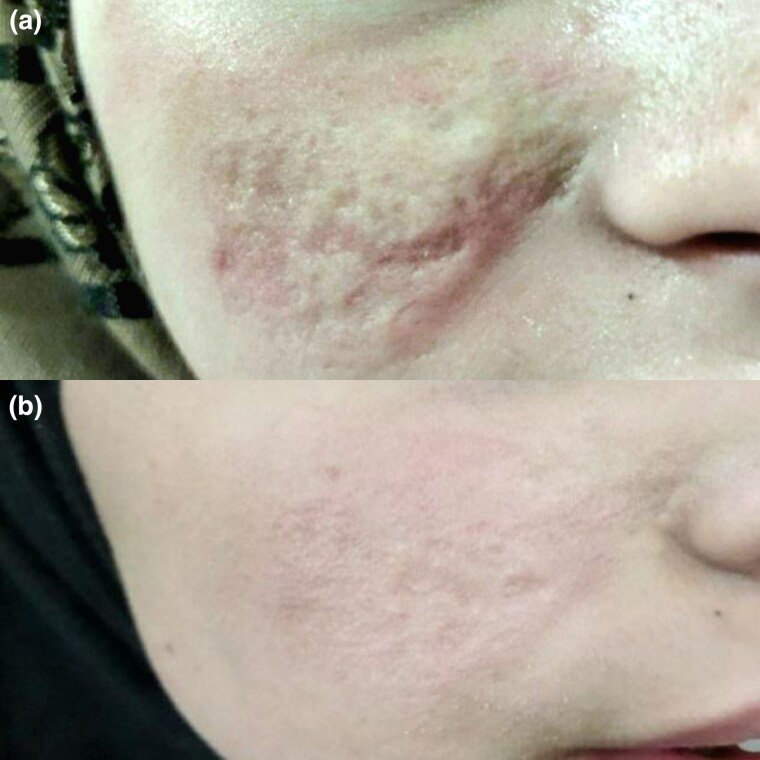
Postleishmaniasis atrophic scars. (a) Before and (b) after 12 months of treatment with topical insulin, a good response was observed.

Overall, 7 of 18 patients (39%) responded well, with 6 (33%) having an average response and 5 (28%) having a poor response. There was a significant difference between the two groups (*P* = 0.001) (Figures 4–6).

### Striae alba scar group (group III)

In subgroup A, one of six patients had a good response (17%), three had an average response (50%) and two had a poor response (33%). In subgroup B, one of the six patients also had a good response (17%), two had an average response (33%) and three had a poor response (50%).

#### Response assessment

Overall, 2 of 12 patients responded well (17%), 5 had an average response (42%) and 5 had a poor response (42%). Patients in subgroup A experienced slightly better results, but the differences were not statistically significant.

### Postoperative scars (group IV)

**Figure 7:**
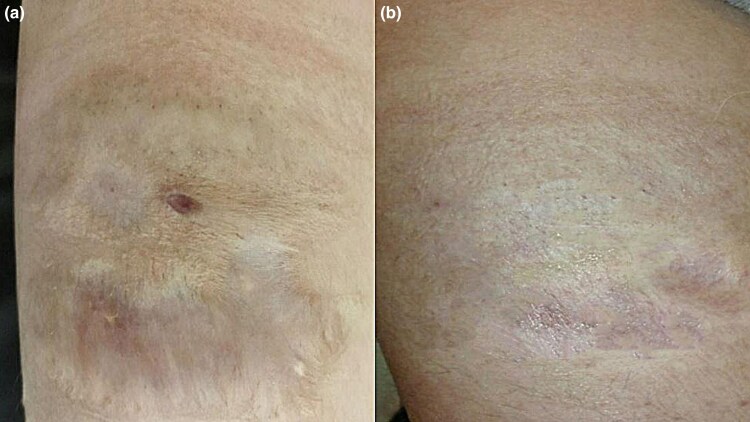
Postoperative atrophic scars. (a) Before and (b) after 12 months of treatment, a good response was observed.

In subgroup A, two of four patients had a good response (50%), one had an average response (25%) and one had a poor response (25%). In subgroup B, one of four patients had a good response (25%), one had an average response (25%) and two had a poor response (50%). Patients in subgroup A experienced slightly better results, but the differences were not statistically significant (Figure 7).

### Patient satisfaction

#### Postacne scars (group I)

In subgroup A, 40 of 100 patients (40%) had a good response, 50 (50%) had an average response and 10 (10%) had a poor response. In subgroup B, 3 of 20 patients (15%) had a good response, 12 (60%) had an average response and 5 (25%) had a poor response. A significant difference between the two groups (*P* = 0.001) was found, indicating that the combination of rapid insulin and microneedling offers a therapeutic advantage for treating atrophic scars due to acne vulgaris compared with microneedling alone.

#### Postleishmaniasis (group II)

In subgroup A, four of nine patients (44.4%) had a good response, four (44%) had an average response and one (11%) had a poor response. In subgroup B, one of nine patients (11%) had a good response, four (44%) had an average response and four (44%) had a poor response. There was a significant difference between the two groups (*P* = 0.001).

#### Striae alba (group III)

In subgroup A, two of the six patients achieved a good response (33%), two achieved an average response (33%) and two achieved a poor response (33%). In subgroup B, one of the six patients also had a good response (17%), two had an average response (33%) and three had a poor response (50%). Subgroup A experienced slightly better results, but the differences were not statistically significant.

#### Postoperative scars (group IV)

In subgroup A, two of four patients had a good response (50%), one had an average response (25%) and one had a poor response (25%). In subgroup B, one of four patients had a good response (25%), one had an average response (25%) and two had a poor response (50%). Subgroup A experienced slightly better results, but the differences were not statistically significant.

### Impact of atrophic scars on daily life

**Figure 8:**
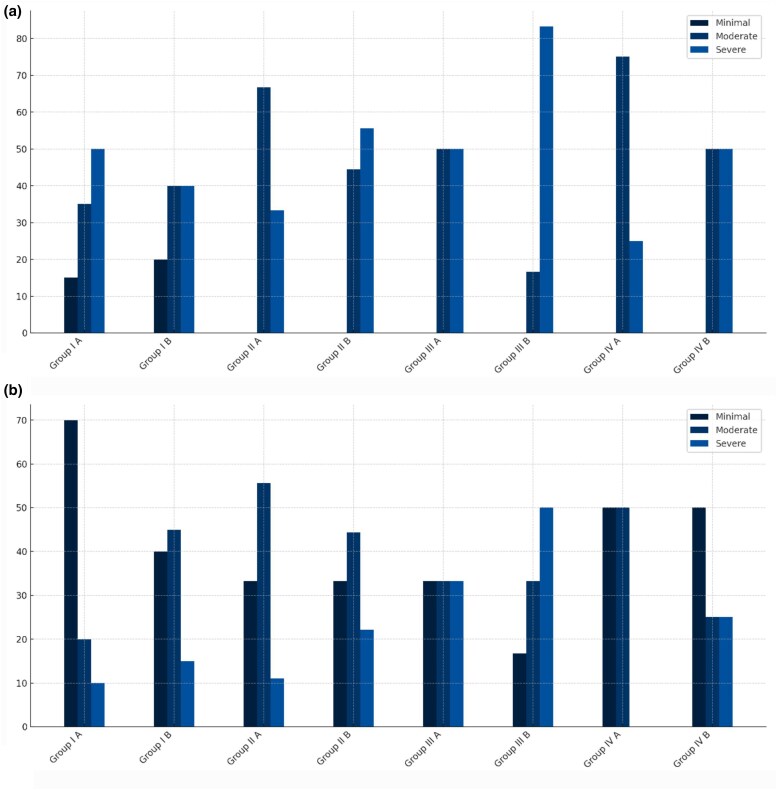
Impact of atrophic scars on daily life. (a) Before and (b) after 12 months of treatment with topical insulin.

Figure 8 compares the impacts before and after treatment. In the postacne group, there was a significant reduction in the impact on daily life between subgroups A and B (*P* = 0.002). However, the results for the other three groups were similar, with no significant differences.

### Analysis of the correlations between treatment outcomes and group I subgroup A characteristics

A comparison of treatment response and age revealed a significant difference, indicating that younger patients, with a mean (SD) age of 22.42 (3.18) years, exhibited good and moderate responses compared with older patients, with a mean (SD) age of 29.34 (3.22) years, who mostly had poor responses (*P* = 0.001). In contrast, no significant difference was observed between sex and treatment outcome (*P* = 0.95). Eighty per cent of the poor response results were associated with a positive family history, which was significantly different (*P* = 0.001). Additionally, patients with previous acne treatments experienced worse results, although this difference was not statistically significant. A prolonged duration without acne treatment was linked to poorer outcomes, with a significant difference noted (*P* = 0.001); specifically, 70% of the poor results occurred in patients who had not received acne treatment for more than 1 year. Older scars were also associated with poorer results (*P* = 0.001). There was no correlation between the results and either the scar site or Fitzpatrick skin type (*P* = 0.40 and *P* = 0.62, respectively). Regarding postacne atrophic scar types, the best results were observed for rolling scars, with a good response rate of 66%. This was followed by boxcar and then icepick, which yielded the worst results (*P* = 0.001).

### Analysis of the correlations between treatment outcomes and group II subgroup A characteristics

A comparison of treatment response and age revealed a significant difference, indicating that younger patients, with a mean (SD) age of 16.22 (4.72) years, exhibited good and moderate responses compared with older patients, who had a mean (SD) age of 25.23 (2.58) years and mostly had poor responses (*P* = 0.002). There was no correlation between response and sex or Fitzpatrick skin type (*P* = 0.10).

### Side-effects

The most common side-effects in both groups included temporary erythema, which lasted a median of 2 days; a burning sensation that typically subsided within a few hours; and dryness, which was effectively managed with moisturizing creams (Table 2). However, persistent erythema lasted for >2 weeks and was observed in two patients in subgroup A (2%). Hyperpigmentation was more common in subgroup A (21%), with no significant difference. It was observed predominantly (100%) in individuals who were exposed to sunlight within 3 days after the session (*P* = 0.001) and was most prevalent in those with Fitzpatrick skin type III (90%), with the remainder in Fitzpatrick IV (*P* = 0.003). This condition was treated with whitening creams. Additionally, hypoglycaemia was reported only in subgroup a (4%), with no significant difference. The patients experienced hypoglycaemic symptoms, as confirmed by their serum glucose levels. These patients adhering to a strict diet. However, their symptoms were alleviated after we administered sugar and advised them to eat before their next session (Table 2).

**Table 2:** Side-effects experienced by patients in the two treatment groups

Side-effect	Microneedling with topical insulin (*n* = 119)	Microneedling with placebo (*n* = 39)	*P*-value
Dryness	119 (100)	39 (100)	
Burning sensation	109 (91.6)	37 (94.9)	0.49
Temporary erythema	119 (100)	39 (100)	
Persistent erythema	2 (1.7)	0	0.55
Miliaria	6 (5.0)	2 (5.1)	0.97
Induced acne	5 (4.2)	0	0.31
Infection	11 (9.2)	3 (7.7)	0.35
Hyperpigmentation	24 (20.2)	5 (12.8)	0.19
Bruises	4 (3.4)	1 (2.6)	0.77
Hypoglycaemia symptoms	5 (4.2)	0	0.31

Data are presented as *n* (%).

## Discussion

TI is gaining popularity in wound healing because of its unique ability to promote tissue repair and regeneration. Its ability to increase cell proliferation, improve angiogenesis and regulate inflammatory responses makes it a valuable option for managing chronic wounds, diabetic ulcers and other skin injuries.^9,10^

Several studies have investigated the efficacy of microneedling combined with TI for treating atrophic scars. One study by Pawar *et al*.^11^ compared the effects of platelet-rich plasma (PRP) and insulin on opposite sides of the face, utilizing a rapid-acting insulin dose of 40 IU. The results indicated that the insulin-treated side experienced a 45% improvement in postacne scars, whereas the PRP-treated side showed a 26% improvement.

In contrast, our study demonstrated a remarkable 90% overall improvement in the postacne scar group with TI, with 50% of patients achieving significant improvement compared with 15% in the placebo group and 40% showing moderate improvement. This enhanced outcome can likely be attributed to several factors, including the inclusion of 12 treatment sessions in our protocol compared with only four in Pawar *et al*.’s study.^11^ This could suggest that a greater number of sessions is associated with more favourable outcomes. A case report detailing a 33-year-old woman with postacne atrophic scars demonstrated an improvement in scar severity, with her Goodman and Baron scale score decreasing from 4 to 3 after four treatments using microneedling with 100 µL mL^−1^. While these results are considered average in comparison with our assessments, it is important to note that we cannot directly compare the two doses due to the limited number of treatment sessions reported in this case.^12^ In a recent study conducted by Mohamed *et al*.,^6^ 30 patients with atrophic facial acne scars were enrolled. Each patient underwent six sessions of microneedling on one side of the face using 1–2 mL of 100 IU mL^−1^, whereas the other side was treated with microneedling combined with hyaluronic acid. There was no significant difference in overall outcomes between the two sides, in contrast to our findings or those of Pawar *et al*.^11^ However, in the present study, 33% of patients reported a good to very good response on the TI-treated side, whereas only 13% in the study by Mohamed *et al*.^6^ reported a good to very good response on the hyaluronic acid-treated side, which matches our results.

In another study on postacne atrophic scars, comparing microneedling combined with TI to microneedling alone, the mean (SD) improvement scores were 10.66 (2.11) and 10.08 (1.88), respectively, with a significant difference (*P* < 0.001), indicating better results in the TI group. These results align with our findings, as we found good results in 50% of patients compared with only 25% in group I, subgroup B. Additionally, in that study, patient satisfaction scores had a mean (SD) value of 6.04 (1.70), which was considered a good response in our TI group, and 5.56 (1.61), which was considered an average response in the non-TI group (*P* < 0.001).^13^

In another study by Mohta *et al*.^14^ a comparison of microneedling combined with autologous PRP with microneedling with TI revealed improvements of 28% and 52%, respectively, with no significant difference found between the two. In our study, there was a significant improvement in the TI group compared with the placebo group. However, the number of sessions (3 in their study vs. 10 in ours) could have influenced the results. However, the author concluded that TI is a superior option due to its ease of use, low cost and noninvasive nature. A study comparing the effects of microneedling and TI with those of vitamin C in treating postacne scars demonstrated a significant difference in outcomes for both treatments. When comparing both TI groups, our study revealed better results, which could be attributed to the number of sessions (4 vs. 10) or the amount used, as they administered 0.1 mL while we used 0.4 mL.^15^

There are few published studies on atrophic scars caused by cutaneous leishmaniasis, largely due to the rarity of this condition in many countries. However, in Syria, it is a prevalent issue affecting individuals of all ages and significantly diminishing their quality of life. A study conducted by AlGhamdi *et al*.^16^ evaluated the effectiveness of CO_2_ fractional laser treatment for these scars and reported an improvement rate of 50%. In contrast, our study achieved an improvement rate of 89%. This discrepancy may be attributed to differences in median age – 17.66 years in our study compared with 32.5 years in theirs – or the number of treatment sessions, which was five in their study. Additionally, it is possible that microneedling combined with TI is more effective for treating these scars than CO_2_ fractional laser therapy is; however, further research with larger sample sizes and consistent methodologies is needed to confirm this. Both treatment modalities resulted in similar side-effects.

In a study comparing microneedling combined with TI at a concentration of 100  IU mL^−1^ with a placebo in the treatment of striae alba, a significant difference was observed between the two treatments. The TI group demonstrated an excellent improvement rate of 70%, whereas the placebo group showed only 30% improvement. In contrast, our study revealed comparable improvement rates between the two treatments, which may be attributed to the lower concentration of TI used in our protocol.^17^

This is the first study to assess this treatment regimen in postoperative atrophic scars. In the TI group, 75% of patients experienced good-to-moderate improvement compared with 50% in the placebo group; however, owing to the small sample size, this result was not statistically significant.

Previous studies have reported similar side-effects. However, most studies did not report hypoglycaemia. A case report revealed a slight reduction in blood sugar levels 120 min after the session, with no symptoms observed.^13^ In our study, five (4%) patients complained of hypoglycaemic symptoms and a reduction in blood sugar levels, which was quickly resolved with sugar intake.

This study demonstrated that microneedling combined with TI is an effective treatment for atrophic scars caused by acne, striae alba, cutaneous leishmaniasis and postoperative wounds, and has minimal side-effects. Additionally, this research highlights the significant impact of atrophic scars on patients’ daily lives and underscores the efficacy of this treatment regimen in improving their quality of life.

One limitation of our study was the small sample size, particularly in the postoperative and striae alba groups. Larger studies are needed to assess the significance of this treatment regimen. Confounders such as the type of previous procedures could not be assessed in our study, which is also a limitation. Another limitation is that the study was not a split-face design, which would have allowed for more precise statistical analyses. Larger prospective cohort studies or randomized controlled trials are needed to evaluate this treatment more thoroughly.

## Data Availability

The data underlying this article will be shared on reasonable request to the corresponding author.
